# Sensitivity of a harbor seal (*Phoca vitulina*) to coherent visual motion in random dot displays

**DOI:** 10.1186/2193-1801-3-688

**Published:** 2014-11-25

**Authors:** Michael Weiffen, Björn Mauck, Guido Dehnhardt, Frederike D Hanke

**Affiliations:** Department of General Zoology & Neurobiology, University of Bochum, ND 6/33, D-44780 Bochum, Germany; Institute for Biosciences, Sensory and Cognitive Ecology, University of Rostock, Albert-Einstein-Str. 3, 18059 Rostock, Germany

**Keywords:** Motion coherence, Global motion, Vision, Motion vision, Random dot display, Pinnipeds

## Abstract

Motion vision is one of the fundamental properties of the visual system and is involved in numerous tasks. Previous work has shown that harbor seals are able to perceive visual motion. Tying in with this experimental finding, we assessed the sensitivity of harbor seals to visual motion using random dot displays. In these random dot displays, either all or a percentage of the dots plotted in the display area move into one direction which is referred to as percent coherence. Using random dot displays allows determining motion sensitivity free from form or position cues. Moreover, when reducing the lifetime of the dots, the experimental subjects need to rely on the global motion over the display area instead of on local motion events, such as the streaks of single dots. For marine mammals, the interpretation of global motion stimuli seems important in the context of locomotion, orientation and foraging. The first experiment required the seal to detect coherent motion directed upwards in one out of two stimulus displays and psychophysical motion coherence detection thresholds were obtained ranging from 5% to 35% coherence. At the beginning of the second experiment, which was conducted to reduce the differential flickering of the motion stimulus as secondary cue, the seal was directly able to transfer from coherent motion detection to a discrimination of coherent motion direction, leftward versus rightward. The seal performed well even when the duration of the local motion event was extremely short in the last experiment, in which noise was programmed as random position noise. Its coherence threshold was determined at 23% coherence in this experiment. This motion sensitivity compares well to the performance of most species tested so far excluding monkeys, humans and cats. To conclude, harbor seals possess an effective global motion processing system. For seals, the interpretation of global and coherent motion might e. g. play a role in the interpretation of optic flow information or when breaking the camouflage of cryptic prey items.

## Background

The ability to see motion is one of the most basic and also one of the most important functions of the visual system. Many adaptive behaviors depend on the detection of motion or the extraction of motion information from a scene (Nakayama^1985^). Motion vision plays a crucial role in depth perception, image segmentation, eye movement control or the perception of moving objects such as predators or prey. Consequently, it does not come as a surprise to find motion vision to be a ubiquitous ability in the animal kingdom. In a previous study, it was shown that harbor seals are able to perceive and stabilize global motion with the help of optokinetic eye movements (Hanke et al.^2008^). Continuing this line of research, we report results of experiments, in which we used underwater projections of moving random dots to investigate the sensitivity of harbor seals (*Phoca vitulina*) to global motion. This study is the first to determine coherent motion detection and coherent motion direction discrimination thresholds in a marine mammal.

**Figure 1:**
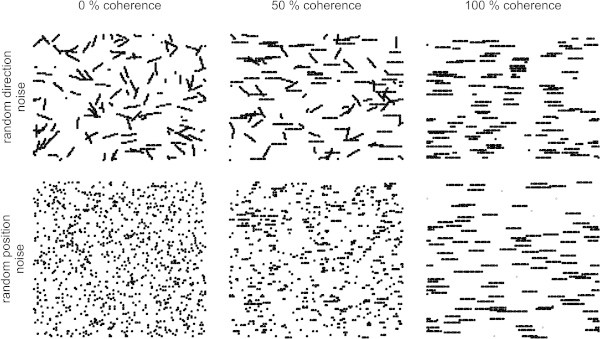
**Random dot displays as used to assess motion sensitivity in a harbor seal.** Schematic representation of random dot displays at different levels of motion coherence (columns) and with either random direction (upper line) or random position noise (lower line). For explanation of random direction and random position noise please see Material and methods. All dots move in different directions at 0% coherence (left). At 50% coherence, one half of the dots move to the right, while the other dots move in random directions (middle). At 100% coherence all dots move to the right (right). Each random dot display as displayed in this figure represents the coordinates of all dots over the time course of 10 frames rendering the trajectories of the dots visible. Please note that, during the coherent motion detection experiment, the coherent signal was directed upwards (not displayed in this figure) whereas, during the coherent motion direction discrimination experiments, it was either leftwards or rightwards.

Random dot displays were used as these displays allow assessing motion sensitivity isolated from form or position cues (see e.g. Morgan and Ward^1980^; Nakayama and Tyler^1981^; Williams and Sekuler^1984^). In such displays, small dots are plotted on a screen with a proportion of these dots, the signal dots, being displaced coherently in consecutive frames by the same distance and in the same direction. Other dots serve as visual noise and are plotted at random positions afresh in each frame or are displaced in random directions (for a review of different types of random dot displays see Scase et al.^1996^). The strength of the motion signal in random dot displays can be easily varied by changing the percentage of signal dots, which is typically referred to as the coherence or correlation of the display. A coherence of 100% means that all dots in the display are signal dots moving in the same direction, while 0% coherence means that all dots in the display are noise dots (Figure 1). Generally, the performance of subjects does not rely on the observation of individual dots but rather on the integration of local motion events into a percept of global motion (Downing and Movshon^1989^; Williams and Sekuler^1984^).

Random dot displays have already been used to assess the sensitivity to detect motion coherence as well as to discriminate coherent motion direction in various species including e.g. pigeons (Bischof et al.^1999^), guppies (Anstis et al.^1998^), rats and mice (Douglas et al.^2006^; Hupfeld and Hoffmann^2006^; Prusky et al.^2001^), ferrets (Hupfeld et al.^2006^), cats (Huxlin and Pasternak^2004^; Pasternak et al.^1995^; Rudolph and Pasternak^1996^), monkeys and humans (see e. g. Newsome and Paré^1988^). Disregarding differences in experimental designs, the lowest coherence thresholds were obtained in monkeys and humans reaching values of 5% or less (see e. g. Newsome and Paré^1988^). Cats can occasionally reach comparably good performances (Rudolph and Pasternak^1996^) but mostly perform worse with thresholds of 20% or higher (Huxlin and Pasternak^2004^; Pasternak et al.^1995^). Thresholds ranging from 20–60% coherence are also typical for the other species tested so far. Thus the wide application of random dot stimuli renders these stimuli valuable for the investigation of motion sensitivity in harbor seals and might allow assessing interspecific differences in sensitivity to global motion with the mentioned limitation resulting from variation in experimental conditions.

Apart from the facts that random dot stimuli offer many advantages in assessing motion sensitivity and that they allow comparison between species, global motion stimuli are most likely of ecological relevance to harbor seals. For harbor seals, the interpretation of global motion might be essential for locomotion and orientation in their environment. Global motion perception plays e. g. an important role in the processing of optic flow, which is elicited on the retina of a seal moving through particulate matter, over the ground or underneath the water surface. It was recently demonstrated that harbor seals are able to perceive optic flow underwater and can accurately depict the simulated heading from an optic flow simulation (Gläser et al.^2014^). Thus seals can possibly benefit from optic flow information for e. g. goal directed locomotion (Warren et al.^2001^), assessing travelled distance (Frenz and Lappe^2005^) or the direction of water flow (Arnold^1974^). Furthermore harbor seals benefit from motion perception while interacting with the environment e.g. for recognizing objects such as prey. Sensitivity to coherent motion would allow determining the direction of movement of a school of fish or could help to detect fish moving in a deviant manner in comparison to the rest of the school with these fishes probably being good targets for prey capture. Furthermore a large portion of the diet of harbor seals consists of flatfish (see e.g. Härkönen^1987^; Härkönen and Heide-Jorgensen^1991^) that are well camouflaged. Sensitivity to coherent motion would enable harbor seals to detect the camouflaged flatfish on the basis of the coherent motion of its parts in line with Lui et al. (2012).

In conclusion, random dot displays provide a powerful tool to assess the sensitivity of the visual system of harbor seals to global image motion. We determined motion coherence thresholds for various stimulus configurations demonstrating that seals possess an effective global motion integration system and that they can achieve at least coherence thresholds comparable to the performances of most terrestrial species examined so far.

## Results

### Acquisition of coherent motion detection during pretraining

**Figure 2:**
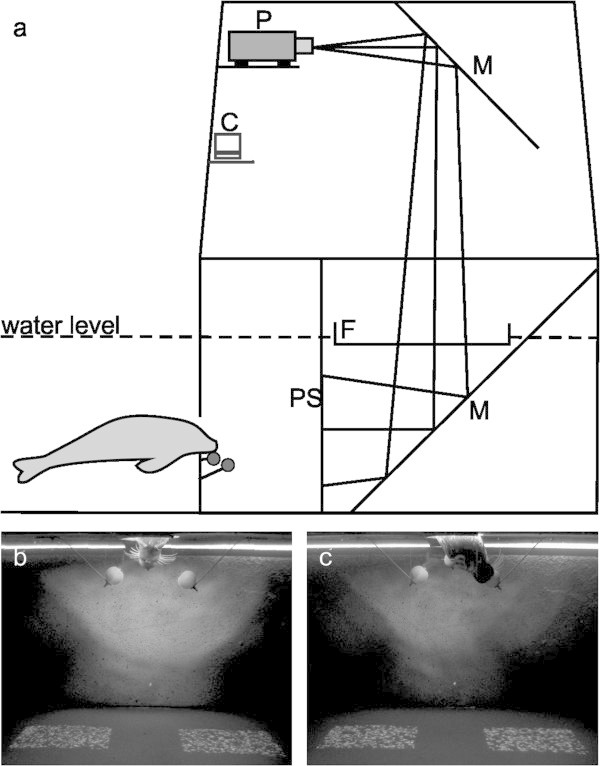
**Experimental setup and procedure. a** Schematic drawing of the projection apparatus depicting the arrangement of projector (P), mirrors (M), frame (F) and projection screen (PS) that was used for the underwater projection. The experimenter sat on the platform above the projection screen out of sight of the experimental animal and operated the computer (C) controlled stimuli. **b** The animal watched the stimulus display by placing its head through a hole in the frontal wall of the experimental chamber resting at a stationary target (view of the experimenter). **c** It communicated its decision, thereby indicating the position of the stimulus area containing the coherent stimulus (coherent motion detection experiment) in case of a correct choice, by moving its head to one out of two response targets (only right response target shown for clarity in A) to the right and left of the stationary target. In the coherent motion direction discrimination experiments (not shown), it indicated the direction of stimulus motion in one stimulus area by again moving its head to one of the two response targets.

During pretraining, the seal had to learn to indicate the position of one of two displays (Figure 2), that contained the coherent motion signal, with a performance of 90% correct choices in three consecutive sessions (see Material and methods). The seal reached the learning criterion after 20 sessions (N = 624 trials). The seal achieved a performance significantly different from chance level in the eighth session (70%, *χ*^2^ = 4.8, p < 0.05) after 264 trials and constantly performed above 75% correct choices from session 12 on. Note that, during pretraining, trial numbers deviating from a multiple of 30 trials result from motivational problems of the experimental animal during some sessions or trials that were added to a standard session due to training aspects.

### Coherent motion detection

**Figure 3:**
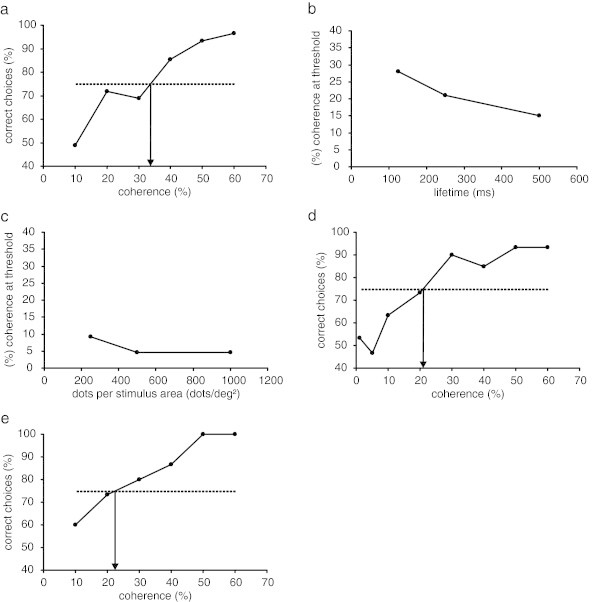
**Sensitivity to coherent motion in random dot displays for a harbor seal.** Results are depicted as psychometric functions **(a** **,** **d** **,** **e)** plotting the performance of the seal in % correct choices as a function of % coherence. Threshold performance of 75% correct choices is indicated by a dashed horizontal line, and arrows pointing to the x-axis mark the thresholds. In **b**, **c** results are plotted as % coherence at the 75% threshold as a function of dot lifetime (in ms) or dot density (in dots/deg^2^). **a** Performance of the seal during the initial determination of coherence threshold (coherent motion detection experiment). Each data point represents the mean performance of 90 presentations; due to human and equipment error the seal’s performance at 20% and 40% coherence was averaged over 89 presentations only. The 75% threshold was determined at 33.7%. **b** Thresholds assessed during the estimation of the effect of dot lifetime (coherent motion detection experiment phase 1). A marked decrease in coherence at threshold with increasing dot lifetime is visible in the performance. **c** Thresholds assessed during the estimation of the effect of dot density (coherent motion detection experiment phase 2). Very low coherence thresholds down to 4.7% coherence were obtained in this experiment, and performance increased with increasing stimulus parameter. **d** Performance of the seal during coherent motion direction discrimination with random direction noise. Each data point represents the mean performance of 30, in the threshold range from 10–40% coherence of 60 presentations. The seal could discriminate motion direction with a threshold of 21.0% coherence. **e** Performance of the sea during coherent motion direction discrimination with random position noise. The 75% threshold for discriminating motion direction with random position noise of 22.5% coherence compares favorably with the direction threshold obtained in the coherent motion direction discrimination experiment with random direction noise.

For initial coherent motion detection threshold determination, the performance of the seal was averaged over 90 presentations after which the performance had stabilized resulting in a clear psychometric function (Figure 3a). The threshold was determined at 34% coherence (Figure 3a, Table 1).

**Table 1:** **Overview of experimental conditions and thresholds obtained in the different experiments**

Exp	Dot size (deg)	Dot density (dots/deg^2^)	Number of dots	Dot lifetime (ms)	Type of noise	Display areas	(%) coherence at threshold
Coherent motion detection experiment							
	0.4	0.15	100	1000	RD	2	33.7
phase 1	0.4	0.15	100	125	RD	2	28.3
	0.4	0.15	100	250	RD	2	21.3
	0.4	0.15	100	500	RD	2	15.0
phase 2	0.2	0.38	250	1000	RD	2	9.2
	0.2	0.77	500	1000	RD	2	4.7
	0.2	1.54	1000	1000	RD	2	4.7
Coherent motion direction discrimination experiment using random direction noise							
	0.2	0.08	500	1000	RD	1	21.0
Coherent motion direction discrimination experiment using random position noise							
	0.2	0.08	500	^a^	RP	1	22.5

Listed are the stimulus configurations used during experiments indicating dot size in deg, dot density in dots/deg^2^, number of dots, dot lifetime in ms, type of noise with RD referring to random direction noise and RP random position noise, and number of display areas together with the coherence thresholds in % coherence obtained for the experimental animal.

^a^lifetime of dots depends on % coherence (see Material and methods).

In the following sessions, coherence detection thresholds with varying parameters were assessed in order to test the influence of these parameters on the seal’s performance within experiments and to assess if the seal’s performance increases with experience over experiments. The seal was trained until reaching a low threshold performance in this coherent motion detection experiment.

In phase 1 of the coherent motion detection experiment, the lifetime of the dots was varied which could help to determine if seals possess an effective local motion integration system. Dot lifetime was decreased to 500 ms, 250 ms and 125 ms, while all other parameters were kept constant (Table 1). Figure 3b gives the resulting coherence thresholds. The seal’s performance improved relative to the initial threshold, resulting in threshold levels below 30% coherence. Coherence thresholds fell with increasing lifetime of the dots (one tailed Spearman Rho r = −1, p < 0.01) from 28% coherence at 125 ms dot lifetime and 21% coherence at 250 ms to 15% coherence at 500 ms (Figure 3b, Table 1).

In phase 2 of the coherent motion detection experiment, dot density was varied in order to test the influence of this parameter on the thresholds. Dot density was changed to 250, 500, and 1000 dots per stimulus display area resulting in dot densities of 0.38 dots/deg^2^, 0.77 dots/deg^2^, and 1.54 dots/deg^2^, while dot lifetime was again set to 1000 ms (Table 1). At these dot densities, the size of the dots was reduced to 0.2 deg to account for the increased number of dots per stimulus display area (Table 1). Figure 3c gives the resulting coherence thresholds. Again the seal showed better performance at these dot densities in comparison to the initial performance at a dot density of 0.15 dots/deg^2^, resulting in thresholds lower than 10% coherence. Overall, the seal performed better the higher the dot density (one tailed Spearman Rho r = −0.866, p = 0.176). The threshold at a density of 0.38 dots/deg^2^ was determined at 9.2% coherence. At dot densities of 0.77 dots/deg^2^ and 1.54 dots/deg^2^, the seal’s coherence thresholds fell to 4.7% coherence (Figure 3c, Table 1).

### Coherent motion direction discrimination with random direction noise

This experiment was conducted in order to eliminate a possible secondary cue that might have guided the seal’s performance in the first experiment, the differential flicker of the positive stimulus. The experimental procedure was changed in a way that now the seal had to discriminate the direction of coherent motion. The seal was viewing one display with either leftward or rightward stimulus motion and had to indicate stimulus motion by moving its head to the left or right respectively. Thus flicker at both sides of the stimulus display area was the same irrespective of stimulus movement. Testing the seal initially with a dot density of 500 dots/stimulus area, a dot lifetime of 1000 ms, and a coherence of 80%, it turned out that the seal was able to adapt to the new experimental condition within one session reaching 86.2% correct choices (*χ*^2^ = 15.2, p < 0.001). In all eight sessions thereafter, the seal’s performance always exceeded 86% correct choices (average performance 90% correct choices, *χ*^2^ = 153.6, p < 0.001). The psychometric function obtained during data collection in this experiment is shown in Figure 3d. Threshold performance was achieved at 21% coherence (Figure 3d, Table 1).

### Coherent motion direction discrimination with random position noise

In this last experiment, local motion events were additionally reduced. These might have guided the seal’s decision in the first two experiments due to the type of noise and the long dot lifetimes chosen in these two experiments. Therefore, we used a display of the random position type in this experiment (Figure 1, Table 1) requiring the seal to respond to no other cue than the global motion of the display. Again the seal had to indicate the direction of the coherent motion signal as in the previous experiment. Using this type of visual noise, the duration and the spatial extent of local motion events is drastically reduced. The seal’s 75% threshold was determined at 22.5% coherence (Figure 3e, Table 1).

## Discussion

### The seal’s performance over the course of the study

The acquisition of the basic motion detection task was fast. The seal reached significant performance already after 264 trials in eight sessions on 5 working days. The individual seal participating in the optic flow study (Gläser et al.^2014^) learned the task involving complex motion stimuli equally fast. In contrast, harbor seals often needed more trials to reach a performance deviating significantly from chance level in visual discrimination experiments. To give an example, it took the experimental animal of this study 600 trials to reach significance level in a visual discrimination task, in which the seal had to discriminate a two rectangle stimulus versus a rectangle triangle stimulus (Hanke et al.^2013^). The fast acquisition in this and the optic flow study (Gläser et al.^2014^) supports the hypothesis that the processing of motion information is of importance for harbor seals (see Global motion).

After the acquisition of the basic discrimination, we assessed coherence thresholds with various stimulus configurations that served to analyze how dot lifetime and dot density affect the seal’s performance within a phase of the coherent motion detection experiment. Dot lifetime was decreased to values below 1000 ms, the dot lifetime that had been programmed during initial threshold determination in the coherent motion detection experiment, thereby reducing local motion events that could guide the seal’s decisions especially with long dot lifetimes. When the lifetime of the dots was set to 125 ms, the seal was still able to detect the coherent motion with a threshold of 28.3% coherence. Instead of relying on long-lived dot streaks, the seal must have integrated motion over a larger area to solve the task. The fact that the performance of seal Henry was affected by variations in dot lifetime indicates that the seal indeed possesses an efficient local motion integration system. However, still the influence of local motion events cannot be denied as the seal’s performance improves the longer the dot lifetime comparable to e. g. humans and pigeons (Bischof et al.^1999^).

A systematic variation of dot density in phase 2 of the coherent motion detection experiment led to an increase in performance of the seal with increasing dot density. This result is in line with previous studies reporting that e. g. global direction discrimination improved as the amount of direction information is increased e. g. by increasing dot density (Watamaniuk^1993^). In phase 2 of the coherent motion detection experiment, the seal even achieved a very low threshold of 4.7% coherence. His coherence thresholds did not further improve, when dot density was increased from 500 dots to 1000 dots per stimulus area, which probably indicates that the seal had reached his personal best performance at least under the respective experimental conditions.

We especially varied stimulus parameters in the coherent motion detection experiment in order to assess if the seal’s performance would increase with experience. Note that learning can only explain the improvement of the seal’s performance documented between but not within experiments as in phase 1 and 2 of the coherent motion detection experiment the stimulus parameter under examination were varied over sessions instead of successively. Indeed, the seal’s thresholds decreased over the whole coherent motion detection experiment. It remains to be answered if this amelioration was due to an optimization of stimulus parameters, reflects a stabilization of performance with time and thus experience as has already been documented for e. g. mice (Douglas et al.^2006^) and monkeys (Britten et al.^1992^) or reflects a learning effect. Over time, the seal might have learnt to pay attention to the differential flickering of the positive stimulus in the coherent motion detection experiment and/or to local motion events. The latter is supported by the results of phase 1 of the coherent motion detection experiment demonstrating an improvement of performance the longer the dot lifetime.

Flickering as a secondary cue was eliminated in the last two experiments, in which the seal was asked to assess the direction of coherent motion, because the flicker was present at both sides of the stimulus and thus should not have provided any information about the motion direction of the signal dots. The seal could directly transfer its experience from coherent motion detection to a discrimination of coherent motion direction within one session. This contradicts the hypothesis that differential flickering had been crucial for decision. However, the resulting threshold is worse than the final thresholds of the coherent motion detection experiment, which could be explained by the absence of flickering, if it had guided the seal’s responses to some extent in the previous experiments. Alternatively, the threshold might be worse in the coherent motion direction discrimination experiment using random direction noise as compared to the best thresholds in the coherent motion detection experiment as the seal had to cope with a totally new experimental situation including a different experimental procedure and direction of stimulus movement. There is evidence from other studies (Britten et al.^1992^) that threshold performance does not necessarily transfer to new experimental conditions.

Local motion events were reduced in the final coherent motion direction discrimination experiment as noise was programmed as random position noise. The seal continued to work with a performance comparable to the previous coherent motion direction discrimination experiment with random direction noise which might indicate that local motion events had not played a crucial role during at least the coherent motion direction discrimination experiment using random direction noise. In our opinion, there was no other possibility than to solve the respective task by the analysis of the global motion of the display, thus by integrating information from a large field of view in this final experiment. It needs to be mentioned that coherent motion direction thresholds might even be lower in harbor seals since training was not continued and thus the seal might have not reached its best threshold performance under the respective experimental conditions. Furthermore, dot density was very low. Therefore, performance thus could potentially increase with a higher dot density, an effect that had been documented in phase 2 of the coherent motion detection experiment.

### Comparison to other species

Generally, comparison to other species is complicated by the fact that the studies conducted with different species differ in the experimental design including variations in threshold criteria, type of noise, direction of movement, dot density and lifetime or size of the display area. The best way to compare motion sensitivity across species is probably to assess motion coherence thresholds in different species in the same study as already done in studies focusing on other species before (Bischof et al.^1999^; Blake and Nawrot^1991^; Newsome and Paré^1988^). Neglecting experimental differences and only focusing on the best performance an animal can achieve, a comparison that was performed in previous reports, the lowest threshold documented for seals in this study compares well with the performance of organisms for which the highest motion sensitivity has been documented before including cats (Rudolph and Pasternak^1996^), humans and monkeys (see e.g. Newsome and Paré^1988^). Thus harbor seals might be able to interpret motion stimuli excellently, which would further underline the significance these stimuli have for harbor seals (see Global motion). In contrast, when setting the focus on the threshold that was obtained in the coherent motion direction discrimination experiment using random position noise which, we think, definitely forced the seal to rely entirely on the global motion of the display instead of on flicker or local motion events, harbor seals achieve a good performance comparable to many other vertebrates (see e. g. Bischof et al.^1999^; Douglas et al.^2006^; Hupfeld et al.^2006^; Huxlin and Pasternak^2004^; Prusky et al.^2001^). To conclude, seals would be equipped with abilities to extract motion from noise generally found in vertebrates although adopting a completely different lifestyle.

### Global motion

Harbor seals seem to have very good access to motion stimuli as demonstrated in this study by the occasionally low thresholds and the fast acquisition of the basic discrimination task. These two phenomena also describe the performance of a harbor seal in a follow up study on optic flow perception (Gläser et al.^2014^). Together with the harbor seal’s well developed optokinetic system (Hanke et al.^2008^), there is thus accumulating evidence that the seal’s visual system is well-adapted to analyze global motion patterns accurately. This might imply that motion information generally and global motion in particular is of importance to harbor seals. Harbor seals can benefit from the analysis of global motion patterns in the context of foraging, locomotion and orientation as already mentioned in Background. In a recently published study, we experimentally demonstrated one context in which seals successfully interpreted a global motion pattern. A harbor seal was able to detect deviations from the simulated heading from an underwater simulation of a forward movement on a straight path through a cloud of dots (Gläser et al.^2014^). With this sensitivity to optic flow as documented in Gläser et al. (2014), harbor seals can probably take advantage of all kinds of optic flow cues. They can profit from optic flow information even and especially under completely turbid conditions in which visual resolution is drastically reduced (Weiffen et al.^2006^) and vision was previously considered to be of no use at all. Many interesting research questions can be derived from the harbor seal’s ability to accurately interpret global motion patterns such as if they can use optic flow information for the integration of a homing vector in terms of path integration (see e. g. Maurer and Séguinot^1995^).

## Conclusions

Harbor seals possess a visual motion analyzing system that enables them to extract coherent motion signals embedded in noise and to determine the direction of coherent motion. The final experiment showed that harbor seals can also globally integrate the coherent motion over the display. Harbor seals probably benefit from their well-developed sensitivity to global motion in the context of underwater prey detection, locomotion and orientation.

## Material and methods

### Experimental subject

The study was conducted at the Marine Science Center, Cologne, Germany. One harbor seal (*Phoca vitulina*; Henry, seven years of age) with previous experience with psychophysical test procedures served as experimental subject. The seal’s daily diet consisted of 2–4 kg of herring supplemented with vitamins and was entirely fed during the experiments. Experiments were conducted in a freshwater pool with a capacity of 300 m^3^ and a maximum depth of 1.7 m. The experiments were carried out in accordance with the current German law on the protection of animals.

### Experimental apparatus and procedure

A chamber (5 m height, 2 m width, 3 m depth) was installed in the experimental pool, which accommodated the test apparatus (Figure 2a), and which was designed for the presentation of computer-generated visual stimuli on an underwater projection screen. Inside the chamber, the light emanating from a projector (Epson EMP-9100, Epson, Suwa, NGN, Japan) was reflected by mirrors twice, passed a rectangular plastic frame with transparent bottom panel, that calmed the water surface, and illuminated the projection screen from behind. The experimental subject could watch the stimulus presentation on the projection screen through a hole in the most frontal wall of the chamber (Figure 2). A central stationing target and two lateral response targets were mounted at the inside of this wall. The experimenter controlled stimulus presentation from a platform above the projection screen and could observe the animal’s behavior through a small hole in the platform. However, the experimenter was completely out of sight of the seal to avoid secondary cueing.

At the beginning of a trial, the seal was required to station in a resting position underwater with its snout in contact to the target in front of the screen (Figure 2a,b). When the seal attended to the screen, the stimulus presentation was started. The duration of the stimulus was about 3 s. However, the seal in most instances answered within the first second of stimulus presentation. The behavioral paradigm, which was used to determine psychophysical thresholds, was a two alternative forced choice procedure. In the first experiment, the seal had to indicate the position of the coherent motion stimulus (positive stimulus) whereas in the following two experiments, it indicated the direction of coherent motion by moving its snout to the respective side. If the seal answered correctly, its response was followed by a piece of cut herring; if it answered incorrectly, the seal’s response was followed by a verbal “no”.

### Stimuli

The stimuli were generated on a PC with a RADEON 9600 graphic card (ATI Technologies Inc.) linked with the projector over a digital visual interface. Random dots displays were designed with a self-written program written in C language in a MS Windows environment using commands of the Open Graphics Library (OpenGL) and the Graphics Library Utility Toolkit (GLUT).

An overview of the stimulus configurations used in experiments, conducted in the order of listing, is depicted in Table 1. Displays consisted of square shaped OpenGL points of two point sizes subtending either 0.4 deg or 0.2 deg of visual angle depending on the experiment. As determined in pretests, the seal was able to detect points of the respective sizes and luminance. The mean luminance of the dots as measured on the projection screen in the empty pool with a luminance meter (Konica-Minolta LS 110) was between 70 cd/m^2^ and 100 cd/m^2^ depending on dot size. The speed of the dots was almost the same in all experiments. However, since we worked with a plane screen and large display areas subtending up to 80 deg of the horizontal visual field in some experiments the speed of the local motion signals varied between 5 deg/s and 10 deg/s depending on their position in the visual field of the animal. The self-written program computed and stored the actual rate at which the frames were rendered by the graphic hardware for adjusting the timing of the stimuli to the frame rate. Correct timing of the displays was confirmed by a photosensitive cell measuring the lifetime of individual dots. Depending on experiment, dot density was varied from 100 to 1000 dots per stimulus display area corresponding to dot densities of 0.15 dots/deg^2^ to 1.54 dots/deg^2^, and dot lifetime was set to 125 ms, 250 ms, 500 ms or 1000 ms.

For training until the seal reached a low threshold performance in the coherent motion detection experiment, the animal was presented simultaneously with two rectangular stimulus display areas of a size of 650 deg^2^ (27 deg × 24 deg) each. One area included a coherent motion signal directed upwards overlaid by visual noise (positive stimulus), whereas the other area only contained visual noise (negative stimulus). Noise was programmed as random direction noise (Figure 1) using the same rule (Scase et al.^1996^). This implies that all dots in the stimulus display areas had the same lifetime and were displaced from frame to frame by the same distance. Signal and noise dots varied in the direction of the displacement; signal dots were displaced only in the upward direction, whereas noise dots were assigned a random direction taken from a direction distribution that was uniform across 360 deg with a step size of 1 deg. The direction was kept constant over the dots’ entire lifetimes. Each individual dot kept its initial assignment as signal or noise dot.

For the first coherent motion direction discrimination experiment, we used the same principal design of motion signal and noise as in the coherent motion detection experiment, however, we eliminated flicker as a secondary cue. In the coherent motion detection experiment, the subject might have not discriminated the stimuli using the pattern of motion signals the stimuli contained but by the pattern of flicker at the edges of the stimulus areas. Since in the positive stimulus of the coherent motion detection experiment the coherent dots left the stimulus area at the top edge and were reintroduced at the bottom edge, the pattern of flicker created by the disappearing and reappearing dots differed between the two stimuli with the amount of flicker in the positive stimulus being higher at the bottom/top edges than at the left/right edges. To eliminate flicker the test situation was changed such that there was only one stimulus area of 6,400 deg^2^ (80 deg × 80 deg) size and the direction of the coherently moving dots was horizontal, either left- or rightwards. Under these conditions, the direction of the coherent motion signal was the only cue for the subjects to rely on, since the pattern of flicker was identical under both stimulus conditions.

To drastically reduce local motion events that might have guided the seal’s behavior due to the noise type and due to the long dot lifetimes mostly programmed in the coherent motion detection experiment as well as the coherent motion direction discrimination experiment using random direction noise, visual noise was additionally programmed as random position noise (Scase et al.^1996^) for the final coherent motion direction discrimination experiment. In the random position display (Figure 1), only the signal dots were displaced and reappeared in the next frame. The noise dots were plotted afresh in each frame at random positions creating local motion signals of varying direction and speed by random pairings of dots in consecutive frames. Since the assignment of a dot as signal dot or noise dot changed from frame to frame, the probability that a signal dot survived *N* consecutive frames was *p*^*N* − 1^, where *p* is the coherence or correlation of the display (Britten et al.^1992^). This way, the average lifetime of the signal dots decreased when the coherence of the display is decreased thereby reducing the spatiotemporal persistence of the local motion event. To create such a random position display, the frame rate of our software was coupled to the vertical refresh rate of our projector (60 Hz) and we calculated the position of the signal and noise dots every second according to the rules described above. Thus, each dot was visible for 1/30 s at a certain position and was then displaced by a fixed distance referring to a signal dot or plotted afresh at a new random position referring to a noise dot.

### Pretraining and data collection

The general experimental procedure was learned by the subject during a pretraining phase that preceded the coherent motion detection experiment. Coherence of the positive stimulus during pretraining was always 100%, which means that all dots of the positive stimulus moved upwards. To facilitate acquisition, the dots, which made up the positive stimulus, were enlarged compared to that of the negative stimulus during the first sessions. In addition, the size and position of the display areas and the dot density was varied until the performance of the animal indicated that it started to respond properly to the stimuli. This procedure resulted in display areas of 650 deg^2^, and 100 dots in each stimulus area corresponding to a density of 0.15 dots/deg^2^. The learning criterion during pretraining was set to a performance of 90% correct choices, which needed to be met in three consecutive sessions in order to assure that the seal was responding on a constant high performance level. No generalization on the parameter coherence was conducted before starting the coherent motion detection experiment.

During experiments, six levels of stimulus intensity were presented in each session chosen to span the prospective threshold range. The experimental animal was presented with more stimuli above the putative threshold than below in order to assure a high motivation level. The position of the positive stimulus was changed from left to right (coherent motion detection experiment) or the direction of motion was varied between rightward and leftward stimulus motion (coherent motion direction discrimination experiments) in pseudorandom order after Gellermann (Gellermann^1933^). In each session, the positive stimulus occurred equally often on the right as well as on the left side (coherent motion detection experiments), rightward and leftward stimulus motion was equally often presented (coherent motion direction discrimination experiments). In the coherent motion detection experiment, in which in one phase dot lifetime and in another phase dot density was varied, dot lifetime and dot density was constant for a session but was varied according to a predetermined schedule over sessions in order to assure that motivation changes of our subjects or training dependent shifts in performance affected all stimulus levels tested in that dimension in similar ways.

Experimental sessions consisted of 36 trials and were preceded by six warm up trials in which the seal was presented with a positive stimulus with 80% coherence three times on the right and three times on the left side. One or two sessions were conducted per day. Psychometric functions were derived from the results of at least five sessions by calculating the mean percentage of correct choices for each stimulus intensity resulting in 30 trials per stimulus intensity. Trial numbers differing from the standard 30 trials per data point are indicated at the respective positions.

The 75% threshold estimates were determined by linear interpolation of the last supra- and first subthreshold value. Statistical evaluation was performed in IBM SPSS Statistics Version 20 (IBM, Armonk, NY, USA).
